# Multiple Roles of dXNP and dADD1—*Drosophila* Orthologs of ATRX Chromatin Remodeler

**DOI:** 10.3390/ijms242216486

**Published:** 2023-11-18

**Authors:** Larisa Melnikova, Anton Golovnin

**Affiliations:** Department of Drosophila Molecular Genetics, Institute of Gene Biology, Russian Academy of Sciences, 34/5 Vavilov St., 119334 Moscow, Russia

**Keywords:** dXNP, dADD1, ATRX, transcription regulation, chromatin insulator

## Abstract

The *Drosophila melanogaster* dADD1 and dXNP proteins are orthologues of the ADD and SNF2 domains of the vertebrate ATRX (Alpha-Thalassemia with mental Retardation X-related) protein. ATRX plays a role in general molecular processes, such as regulating chromatin status and gene expression, while dADD1 and dXNP have similar functions in the *Drosophila* genome. Both ATRX and dADD1/dXNP interact with various protein partners and participate in various regulatory complexes. Disruption of ATRX expression in humans leads to the development of α-thalassemia and cancer, especially glioma. However, the mechanisms that allow ATRX to regulate various cellular processes are poorly understood. Studying the functioning of dADD1/dXNP in the *Drosophila* model may contribute to understanding the mechanisms underlying the multifunctional action of ATRX and its connection with various cellular processes. This review provides a brief overview of the currently available information in mammals and *Drosophila* regarding the roles of ATRX, dXNP, and dADD1. It discusses possible mechanisms of action of complexes involving these proteins.

## 1. Introduction

Histones and accessory proteins such as histone-modifying complexes, chromatin regulators, and transcription factors are essential for maintaining chromatin structure and regulating gene expression. The first level of chromatin compaction is the nucleosome, which consists of histone protein octamers with DNA wrapped around them twice superhelically [1]. Nucleosomes provide an efficient mechanism for compacting eukaryotic genomes for their storage within the nucleus. However, they also build a significant barrier to factors involved in general genetic processes. The interaction between DNA and the nucleosome is dynamic, providing for nucleosome removal from DNA or making nucleosomes more loosened and allowing regulatory proteins access to DNA [2]. Chromatin remodeling factors are indispensable in regulating such events as nucleosome assembly and remodeling, the covalent modification of histone proteins, and the targeted incorporation of different histone variants [3].

Two classes of multi-subunit complexes control chromatin remodeling: one type can covalently modify histones and DNA; a second group uses ATP (adenosine triphosphate) energy to mobilize nucleosomes [4]. ATP-dependent remodeling complexes are highly conserved from yeast to humans [5,6,7,8,9].

In this review, we focus on the *Drosophila* proteins dXNP and dADD1 and their human ortholog ATRX. The ATRX and dXNP proteins belong to the SNF2 protein family since they contain special domains: the SNF2 family domain and the helicase-like ATPase domain. SNF2 family proteins (named after the founding sucrose nonfermenting 2 protein in *S. cerevisiae*) are not real helicases, as they lack the ability to separate nucleic acid strands. SNF2 proteins are DNA translocases that apply an ATP-dependent torsional strain to DNA, providing the necessary force to remodel nucleosomes or, in some cases, other DNA-protein complexes [10,11,12].

The ATRX protein plays a significant role in maintaining genome stability. ATRX regulates fundamental mechanisms of cell life, such as repression of heterochromatic regions, resolution of secondary structures, expression of specific genomic loci, double-strand break repair, and aging [13,14,15]. The role of ATRX is context-dependent as it promotes gene expression at specific loci, such as the α-globin locus, while at other genomic regions—such as telomeres, centromeres, or the X chromosome—it maintains chromatin repression [8,16,17,18,19]. However, despite the large amount of accumulated data, our understanding of the molecular mechanism of ATRX action remains limited.

Here, we compare the roles of ATRX in the functioning of mammalian genomes and comparable roles of dXNP and dADD proteins in *Drosophila* genomes. In addition, we review the molecular interactors of these proteins and discuss possible mechanisms of action of complexes involving dXNP, dADD, and ATRX.

## 2. Structure and Nuclear Localization of the ATRX, dXNP, and dADD Proteins

The human *ATRX* gene was described about 20 years ago as the main gene in which mutations cause ATRX syndrome. *ATRX* gene mutations manifest in several phenotypic features, including mental retardation, craniofacial and urogenital deformities, psychomotor failure, and alpha thalassemia [13,14,15].

**Figure 1:**
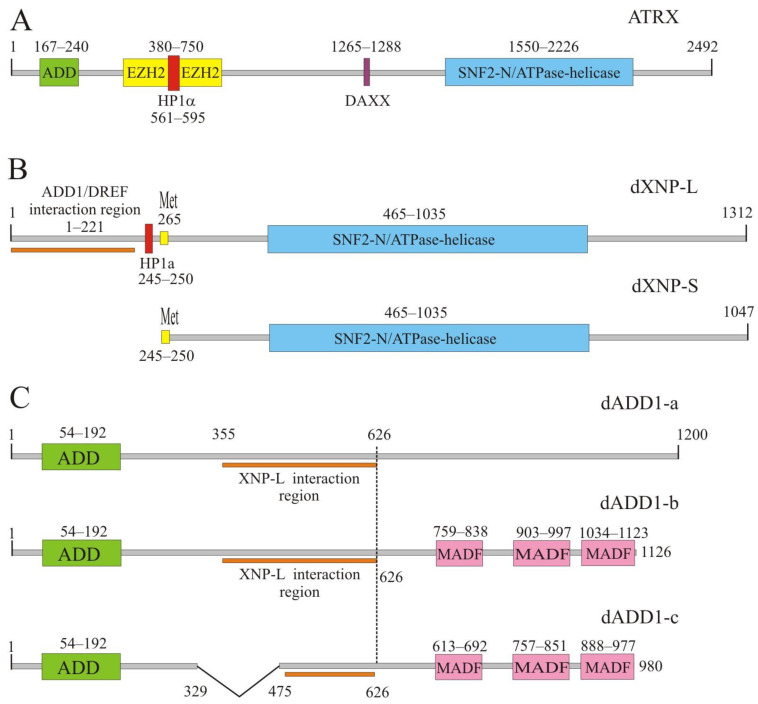
Schematic representation of protein domains. Numbers indicate amino acid position. Borders of domains are indicated above the schemes. (**A**) ATRX protein. DAXX represents DAXX protein interaction motive. (**B**) dXNP protein. XNP-L and XNP-S isoforms indicated. The small isoform (dXNP-S) is generated due to alternative translation because of presence of the second start codon at 265 aa. (Indicated as Met “265”). (**C**) dADD1 protein isoforms. The punctuated vertical line shows the border of the common part of all isoforms.

At the N-termini of ATRX protein is found the ADD domain (named for the three proteins containing this domain: ATRX, DNMT3b, and DNMT3L), which consists of PHD and GATA-like zinc fingers. At the C-terminus of ATRX, there is an SNF2-like helicase/ATPase domain (Figure 1A). Pathologies described in patients were equally likely to occur with mutations in the ADD and helicase/ATPase domains [20]. It has been demonstrated, using a two-hybrid yeast assay, that the region of the ATRX protein located between the ADD and the helicase/ATPase domain can interact with the repression proteins HP1α (heterochromatin protein1 α) and EZH2 (Enhancer of Zeste Homolog 2) from the Polycomb group and histone chaperone DAXX (Death Domain Associated Protein) (Figure 1A) [21,22,23,24]. The ATRX protein is predominantly localized in pericentric heterochromatin, telomeres, and the inactivated X chromosome, suggesting its involvement in maintaining the repression status of chromatin [25,26,27].

In higher eukaryotes, the ATRX protein is highly conserved. However, in *Drosophila*, the two main ATRX domains, dADD and helicase/ATPase, are encoded by different proteins, dADD1 and dXNP, respectively [28,29,30]. Simultaneous mutations in genes encoding dXNP and dADD1 proteins cause the formation of melanomas and a significant (up to 50%) decrease in survival [28]. Thus, the joint work of the dADD1 and dXNP proteins in *Drosophila* is as necessary as the intact ATRX protein in mammals.

There are two isoforms of the dXNP protein carrying the helicase/ATPase domain, the large (150 kDa) dXNP-L and small (120 kDa) dXNP-S (Figure 1B). The small isoform is generated due to the presence in the protein of the second start codon at 266 a.o. [29]. dXNP-L is predominantly localized in pericentromeric heterochromatin, while dXNP-S is localized in euchromatin regions [28,29]. However, the dXNP-L protein is not the main factor of heterochromatin since it is mainly localized in the cell as a separate focus corresponding to a block of decondensed transcribed satellite repeats located near pericentromeric chromatin. It is noteworthy that, in experiments using antibodies common to both isoforms, the dXNP protein distributed over polytene chromosomes outside the pericentromeric region has good co-localization with the Pol II elongation form [31]. This indicates the involvement of dXNP in the transcription process.

The *Drosophila* dADD1 protein has three isoforms resulting from alternative splicing. All isoforms contain an ADD domain but differ in C-termini: two carry a three-fold repeated MADF domain (myb/Sant-like domain in Adf1) (Figure 1C). The MADF domain can bind to repetitive DNA sequences [32] or may be involved in protein-protein interactions [33]. Isoforms dADD1a and dADD1b interact by their common region with 1–122 aa of the dXNP-L isoform [28]. On polytene chromosomes, the ADD1 proteins are significantly colocalized with the dXNP-L protein, not only in the pericentromeric chromatin region but also in the euchromatin region. Nevertheless, independent bands for both dADD proteins and dXNP-L were observed along chromosome arms, suggesting independent binding of these proteins to chromatin in some cases [28].

## 3. ATRX and dXNP Proteins Regulate Chromatin Status through H3.3 Histone Deposition

In addition to the canonical core histones, cells express nonallelic-isoform histone variants [34]. Canonical histones are expressed during the S phase of the cell cycle and are incorporated into chromatin in a DNA replication-dependent manner, while replication-independent histone variants are expressed and incorporated throughout the cell cycle [35]. Histone variants are an essential regulatory tool for maintaining chromatin structure and are involved in multiple processes, including chromatin stability, DNA repair, and transcriptional regulation. One of the best-studied histone variants is H3.3, which can replace canonical H3 histone. It is believed that the H3.3 variant is a marker of active chromatin, as H3.3 deposition generally indicates dynamic chromatin, with enriched regions exhibiting high turnover rates [36]. H3.3 deposition is mediated by two kinds of chaperones: HIRA (histone regulator A) and DAXX. The HIRA complex deposits the H3.3 histone variant in euchromatin gene-rich regions, while DAXX is involved in deposition within genome regions enriched in repeated sequences, mobile elements, telomeres, and pericentromeric regions [37,38]. The ATRX protein has been shown to interact directly with DAXX, and the structure of this interaction has been demonstrated [24]. 

In telomere regions and pericentromeric chromatin, the ATRX/DAXX complex replaces the H3 histone variant with H3.3 through ATRX helicase/ATPase activity [7,39,40]. Unlike the DAXX chaperone, HIRA is not required for ATRX activity in the deposition of H3.3 histone in euchromatic regions [7]. It has been shown that, in individual cells, untranscribed inducible multicopy transgenes colocalized with ATRX and DAXX proteins. However, both proteins disappeared from the transgenes upon induction of transcription. In addition, in a cell line with reduced ATRX gene transcription levels, exogenous expression of ATRX resulted in repression of multicopy transgenes transcription. These data confirm that the ATRX and DAXX proteins play a role in forming repression chromatin [41,42]. 

**Figure 2:**
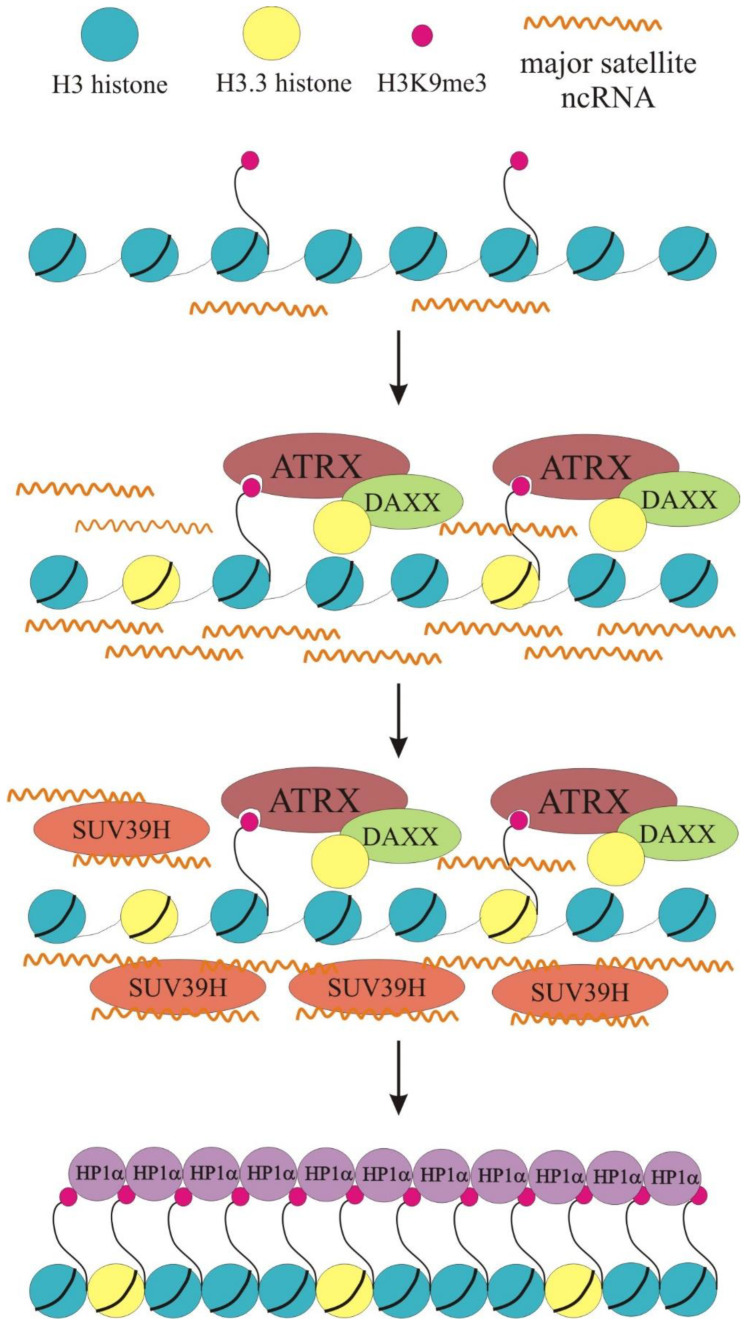
Pericentromeric chromatin establishment and maintenance are mediated by the ATRX-DAXX complex. At pericentromeric chromatin, the ATRX-DAXX complex recognizes H3K9me3 and promotes H3.3 histone deposition. This stimulates the transcription of small noncoding RNAs, which, along with H3K9me3, recruit SUV39H methyltransferase. SUV39H specifically tri-methylate lysine 9 of histone 3, and this marker is recognized by HP1α repressive protein.

The introduction of histone variant H3.3 is predominantly marked by active (enhancers, promoters, gene bodies) “open” chromatin [43,44]. This seems incongruous with the observed location of H3.3 histone in pericentromeric heterochromatin and telomeres [7]. Moreover, it was found that ATRX is present on pericentromeric DNA repeats together with DAXX and that the reduction in ATRX expression affects both the transcription and DAXX-dependent deposition of H3.3 onto these repeats [38]. Thus, it can be assumed that the ATRX/DAXX-dependent administration of histone H3.3 in the pericentromeric chromatin can contribute to the maintenance of “open” chromatin and, therefore, active transcription of pericentromeric repeats (Figure 2). The resulting multiple transcripts of pericentromeric repeats stimulate the formation of the repression status of pericentromeric chromatin. It is possible that recruitment of ATRX/DAXX to the pericentromeric region occurs by recognition of H3K9me3 marks. It is unlikely that H3K9me3 binding alone targets SUV39H, for many H3K9me3 sites exist in the mammalian genome, but not all recruit SUV39H [45]. There must be additional mechanisms to effectively recruit proteins providing a heterochromatic state of chromatin. Indeed, it was demonstrated that both RNA and H3K9me3 act together as main determinants of SUV39H localization to pericentromeric chromatin [46,47,48]. Therefore, ATRX/DAXX stimulates the transcription of RNA, which, along with H3K9me3, recruits SUV39H methyltransferase. SUV39H in turn, specifically tri-methylate lysine 9 of histone 3, and this marker is recognized by HP1 repressive protein.

It has been shown that inactivation of the ATRX protein leads to a global increase in the incorporation of the transcriptional repression marker—the macro-H2A variant of histone—into the genes of the α-globin cluster, which results in a significant decrease in α-globin gene expression [8]. Based on this, it can be assumed that the ATRX protein replaces the histone macro-H2A variant with H2A and, therefore, participates in forming active chromatin. Interestingly, ATRX acts independently of the DAXX protein when substituted with macro-H2A. All these data indicate that ATRX maintains a balance between chromatin’s active and repressive states.

In *Drosophila*, orthologs of DAXX–DLP (DAXX-like protein) and HIRA have been identified [49,50,51]. Like its mammalian ortholog, DLP deposits H3.3 histone in pericentric chromatin and telomeres with dXNP assistance. Loss of dXNP function is characterized by reduced viability, which is further reduced when DLP function is reduced in parallel. This indicates that dXNP and DLP may functionally cooperate in *Drosophila* as ATRX/DAXX do in mammals. 

In euchromatin, dXNP and HIRA associate with genomic regions where nucleosomes are unstable or disassembled and DNA is exposed. Both dXNP or HIRA single mutants have limited phenotypes and weakly affect H3.3 deposition, implying they have redundant functions. However, double mutants of these factors strongly reduce the enrichment of H3.3 histone variants along chromosome arms [52]. This observation implies the existence of two distinct pathways during H3.3 nucleosome assembly: one mediated by HIRA and the other mediated by dXNP. Moreover, an independent dXNP pathway was confirmed by the observation that H3.3 incorporation was only slightly reduced in flies lacking DLP or both HIRA and DLP [49]. Hence, it is possible that the independent dXNP pathway is always functional, although less efficient than the DLP or HIRA-associated pathways.

**Figure 3:**
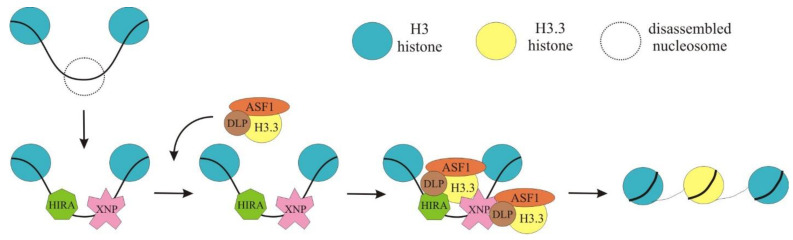
DLP containing the H3.3/H4 predeposition complex is recruited at chromatin with unstable or disassembled nucleosomes by dXNP/HIRA. When chromatin contains exposed regions with disassembled nucleosomes, HIRA and dXNP recognize and bind exposed DNA at chromatin gaps. A separate predeposition complex containing DLP, ASF1, and the H3.3/H4 heterodimer is then recruited to chromatin-bound dXNP and/or HIRA. Finally, dXNP and Hira capture histones from ASF1-DLP and wrap them with DNA to rebuild new nucleosomes.

ASF1 (anti-silencing factor 1) is a general histone chaperone that is associated with new histone dimers in the cytoplasm and escorts them into the nucleus [53]. It has been hypothesized that a separate predeposition complex, containing ASF1 and H3.3/H4 heterodimers, is recruited by dXNP and HIRA and that the heterodimers are subsequently incorporated into new nucleosomes. Thus, dXNP serves as a binding platform for the recruitment of H3.3 predeposition complexes to chromatin gaps. Such complexes are believed to contain H3.3-H4 heterodimers, ASF1, and additional factors [52]. Moreover, the chaperone DLP has been shown to interact directly with ASF1, suggesting their co-recruitment to exposed DNA by dXNP (Figure 3) [49].

## 4. Dual Role of dADD1, dXNP, and ATRX in the Regulation of Transcription

In experiments with purification of the HP1a protein complex followed by MALDI analysis, it was found that dADD1 and dXNP proteins are the main partners of the HP1a repression protein [30]. A decrease in the ATPase activity of dXNP, as well as a decrease in the HP1a level, led to a disturbance of the chromosome structure. On the contrary, when dXNP was overexpressed, the chromatin of polytene chromosomes compacted, and the number of bands increased [54]. Only the dXNP-L isoform has consensus for HP1a binding, and disruption of this motive leads not only to the abolishment of dXNP-L–HP1a interaction but also to the relocalization of dXNP-L from the pericentromeric region of polytene chromosomes to euchromatic regions [29].

It has been shown that, in contrast to dXNP-S, the dXNP-L isoform interacts with the DREF activator protein [9,28]. Interestingly, the dADD1 interaction region of dXNP-L overlaps with dDREF but not the HP1a interaction region. Thus, dADD1 and DREF may compete for dXNP-L binding. It can be assumed that dXNP-L may be recruited to chromatin through dADD1 or DREF and is involved in the functioning of various complexes. For example, it has been shown that dXNP can suppress the transcription of some DREF-dependent genes (*pnr*, *osa*, and *E2F*), but not others (*PCNA* and *rp49*) [9]. In addition, in genetic experiments on the *bw^D^* and *In(w)^m4h^* model systems, a strong suppression of position effect variegation (PEV) was observed, both with a decrease in expression and with overexpression of the dXNP protein [31]. Thus, dXNP may maintain a balance between chromatin’s active and repressive states and act as a promoter-specific regulator of gene expression. A mutation affecting a region common to all dADD1 isoforms weakened the PEV-dependent repression of the model gene [28,30].

Analysis of heterochromatic gene occupancy by dADD1 revealed an increase in binding at the TSS of moderate to highly expressing genes. The subset of these tested heterochromatic genes lowers the expression in the dADD1 RNAi background, while others were not sensitive or up-regulated [55]. This was confirmed by experiments in polytene chromosomes, where overexpression and inactivation of dADD1 affected the expression of the heterochromatic genes [54].

Previously, strong interactors of dADD1 have been identified, such as methyltransferases Su(var)3–9 and Eggless/dSetDB1 [30]. Su(var)3-9 is a repressor located predominantly in the pericentric heterochromatin and is indispensable for the methylation of H3K9 in the nucleosomes associated with repeated DNA sequences. dSetDB1 contributes to female germline cell differentiation and silencing of transposable elements, as well as to maintaining methylation of H3K9 at the fourth *Drosophila* heterochromatic chromosome [56,57]. Nevertheless, it was recently described that, in somatic cells, specifically in polytene chromosomes, dSetDB1 is present mainly in euchromatin, where it appears to contribute to transcription initiation and formation of chromatin domain borders, and that minimal dSetDB1 binding is detectable in the pericentric heterochromatin [58,59,60]. 

Constitutive overexpression of all dADD1 isoforms is lethal in the early stages of development. Overexpression of dADD1 in polytene chromosomes changes the chromatin structure: due to decompaction and loosening of chromatin fibrils in the centromeric region, the band-interband structure is partially lost [54]. This phenotype is most pronounced upon overexpression of the dADD1-a isoform. In addition, overexpression of dADD1 isoforms leads to the delocalization of HP1a, dXNP proteins, and dADD1 isoforms themselves, and decreases the level of histone variant H3K9me3. Thus, it is possible that overexpression of dADD1 proteins in polytene chromosomes may disrupt equilibrium in the dADD1/dXNP/HP1/Su(var)3-9 protein complex at pericentric heterochromatin or the dADD1/dXNP/HP/(EGG/dSETDB1) protein complex in euchromatin domain borders, resulting in observed chromosome abnormalities. It has been shown that both the overexpression and inactivation of individual components of these complexes significantly change the expression, not only of hetero but also of euchromatin loci [9,30,54,61].

In mammals, the ATRX and DAXX proteins deposit H3.3 on retroviral elements [62,63,64]. Silencing of retrotransposons involves the establishment of a heterochromatic region covering the regulatory sequences of these elements. This heterochromatic structure is very similar to that of pericentric heterochromatin, enriched in modification marks such as H3K9me3 and H4K20me3. In contrast to pericentric chromatin, H3K9me3 signatures on retrotransposons are predominantly established by SetDB1 histone methyltransferase and not by Su(var)3-9 [62]. In heterochromatin formation, the H3K9me3 mark recruits HP1 proteins with high efficiency. Striking correlations were observed in retrotransposon occupation between the H3.3 histone variant H3K9me3 and the silencing factors Trim28 and SetDB1 [63,65]. Depletion of H3.3 led to reduced levels of Trim28, DAXX, and H3K9me3 on retroviral elements and the deregulation of nearby genes [62,63]. Thus, through the ATRX–DAXX-deposition pathway, the H3.3 histone variant may play a significant role in retroviral elements heterochromatin maintenance.

At the same time, recent ChIP-Seq studies have identified multiple euchromatic targets, such as enhancers, promoters, and actively transcribed genes regulated by ATRX. The presence of ATRX at euchromatic sites was positively correlated with the level of histone H3.3 and the expression level of these genes [16]. Thus, by maintaining the presence or location of the labile variant of histone H3.3 in active genes and regulatory elements, ATRX may contribute to creating “open” chromatin accessible to transcriptional factors.

## 5. Role of ATRX in Maintaining Mammalian Telomere Stability

**Figure 4:**
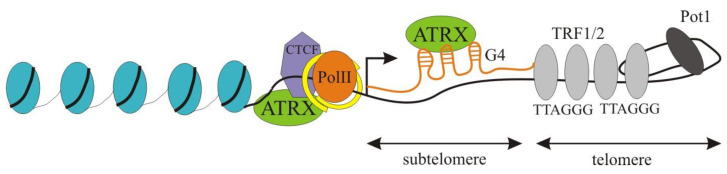
Scheme of mammalian telomere. Nucleosomes are represented as blue ovals with DNA (black line) wrapped around them. ATRX, CTCF, and PolII are indicated. Cohesin is represented by the yellow ring. Long noncoding RNA TERRA is represented by the orange line, with hairpins representing G-quadruplexes (G4). Grey ovals represent the TRF1 and TRF2 proteins, bound to double-stranded DNA with TTAGGG repeats. The black oval represents POT1-TPP1, a subunit of shelterin, that binds to the telomeric overhang.

Telomeres are DNA/RNA/protein complexes at the ends of eukaryotic chromosomes that prevent the ends of the chromosome from degradation, end-to-end fusion, or improper recombination [66,67,68]. Vertebrate telomeric DNA comprises multiple TTAGGG repeats packaged in a well-described protein structure called shelterin [69]. Adjacent to the TTAGGG are variable tandem repeats known as the subtelomeres. They are bound by different proteins that have been implicated in the regulation of transcription and chromatin structure [70]. A long noncoding RNA named TERRA (TElomeric Repeat-containing RNA) is transcribed from subtelomeres, which interacts with telomeres through the formation of DNA: RNA hybrids, R-Loops and G-quadruplex structures (Figure 4). Typically, telomerase elongates the telomeric repeats in germ cells—embryonic and adult stem cells—but not in somatic cells [67]. In cancer, telomeres are elongated by two mechanisms: (1) activating the expression of the catalytic subunit of telomerase, hTERT, (telomerase positive), or (2) maintaining telomeres by an ALT (alternative lengthening of telomeres) pathway that utilizes homolog recombination at telomeric regions [71].

ATRX localizes to the subtelomeric regions of the chromosomes in telomerase-positive human cells, as confirmed by FISH and ChiP analysis (Figure 4). Interestingly, ATRX peak enrichment occurred in the subtelomere at ~1.1 kb from the terminal repeats, which strongly overlaps with the CTCF (CTC binding factor) binding region [72]. It has been shown that in telomerase-positive cells, the presence of ATRX at telomeres provides cell cycle-dependent regulation of TERRA expression. In interphase, ATRX inactivation leads to a decrease in TERRA levels and a decrease in the recruitment of RNAP II and SMC1/cohesin in subtelomeric regions [72]. Inactivation of ATRX leads to increased TERRA levels in the G/M phase, where it usually reduces [73]. These results suggest that ATRX downregulates TERRA in the G2/M phase of the cell cycle. The same increase in TERRA transcription in the G/M phase is typical for ALT cells. Recent studies highlight a strong correlation between the occurrence of the ALT pathway and the presence of mutations in the *ATRX* gene. A possible role of ATRX in ALT cells is the removal of replication protein A (RPA) from telomeres after the replication phase to prevent unwanted recombination or sticking of sister chromatids [73,74]. Another opportunity for ATRX to influence telomere stability in ALT cells is its ability to bind and regulate R-loop and G-quadruplex structure formation. Considering that telomeric R-loops are one of the substrates for homologous recombination in ALT, ATRX may prevent the triggering of ALT by suppressing these recombinations [75]. In hTERT cells, G-quadruplexes formed by the TERRA transcript can interact with ATRX and dose its binding to subtelomeric repeats, thus regulating the expression of TERRA itself [76]. This feedback system apparently allows fine regulation of the chromatin status of telomeres.

However, the exact chromatin status of human telomeres remains unclear. It is assumed that telomere chromatin is not spatially homogeneous: the proximal part is more heterochromatic due to its adjustment to the subtelomere, while the distal part is marked by active histones and compacted by shelterins [77]. A sharp decrease in nucleosome density is observed in telomeres under ATRX inactivation [78]. It was recently shown that PRC2 (Polycomb Responsible Complex 2) mediates H3K27me3 at telomeres, that this process is dependent on TERRA, and that it has a positive effect on the recruitment of H3K9me3, H4K20me3, and HP1 to telomeres [79]. Interestingly, PRC2 recruitment is dependent on the RNA-binding activity of ATRX [19]. Furthermore, a decrease in the H3K9me3 mark, due to the suppression of SUV39H1/2 activity, leads to increased binding of ATRX to telomeres [75]. Based on these data, it can be assumed that, in contrast to pericentric heterochromatin, ATRX is recruited in telomeres not by the H3K9me3 mark, but due to binding to TERRA. Such binding may stimulate the recruitment of repressive marks to telomeres to balance existing active marks [79] and maintain proper telomere chromatin status.

## 6. The Role of dADD1 and dXNP in the Regulation of Transcription and Integration of Retrotransposons in *Drosophila* Telomeres

**Figure 5:**
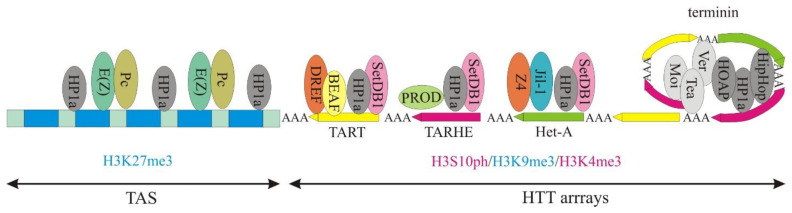
Scheme of *Drosophila* telomere. Proteins involved in chromatin organization are indicated. To the right is the capping complex, named terminin, and t-loop structure. Dark grey ovals represent proteins binding to double-stranded DNA, while pale gray ovals denote proteins that bind to single-stranded DNA. Prevailing histone modification for each region is indicated below, with active marks denoted in red and repressive marks in blue.

*Drosophila* telomeres have a principally different structure (Figure 5). Nevertheless, like a human telomere, the very end of the chromosome contains a capping structure named terminin, which is comprised of proteins interacting with double-stranded (HP1a, HOAP, HipHop) and single-stranded (Moi, Ver, Tea) DNA. Both these types of capping proteins are involved in t-loop formation [80]. Mutations in genes encoding these proteins result in frequent telomere fusions, phenotypes that determine the participation of the protein in the capping structure. Next (in the centromeric direction) are tandem repeats built from orientated head-to-tail non-LTR retrotransposons (Het-A, TART, TARHE), hence its name HTT. Transcription of these retrotransposons provides elongation of chromosomes, which are shortened during each cell division. The retrotransposition mechanism must be active in germ cells but silenced in somatic cells, in order to maintain chromosomal stability [81]. Telomere function and stability require establishing an unusual epigenetic pattern at HTT, which organizes specific chromatin classes with mixed characteristics of heterochromatin and euchromatin [82,83]. Mutations in the genes that code for proteins responsible for maintaining these chromatins result in changes in retrotransposon expression and increased telomere length [84,85,86].

Telomere-associated sequences (TAS) are subtelomeric satellite repeats [81]. It has been demonstrated that HP1 is present along the HTT array, as well as in TAS, and acts as a negative regulator of transcription of telomeric retroelements (Figure 5) [87,88]. The main proteins associated with telomeres were identified by PICh assay (proteomic analysis of isolated chromatin segments); this detected, among other important proteins, both dXNP and dADD1 [89]. 

Given that ATRX affects the integrity of mammalian telomeres, it can be assumed that *Drosophila* orthologs dXNP and dADD1 may also play a role in telomere stability. Indeed, an increased number of telomeric fusions was detected in flies with combined null *add1* mutation (*add1*^2^/*add1*^2^) and the *xnp*^2^ allele (*xnp*^2^/+), which affects both dXNP isoforms. The combination of null *add1* mutation with the *xnp^3^* allele, which affects only the dXNP-L isoform, showed no increase in telomeric fusions [61]. These data suggest that only the dXNP-S isoform has an important role in preventing these types of chromosomal aberrations.

In flies with only the *add1*^2^/*add1*^2^ genotype, the TART retrotransposon demonstrates a higher number of transcripts but a lower number of integrated copies, while transcription and integration of the HeT-A element did not alter significantly. Flies with the *add1*^2^/*add1*^2^;*xnp*^2^/+ combination demonstrates more TART and HeT-A retrotransposon transcripts and a higher number of their integrated copies [61]. These results suggest that, for the TART element, dXNP is necessary to prevent the integration of the retrotransposon copies, while dADD1 is responsible for its transcript repression. It assumes that homologous recombination and gene conversion are additional mechanisms in *Drosophila* for maintaining telomere length [90]. It is possible that dXNP controls the frequency of homologous recombination in telomeres as vertebrate ATRX does to prevent ALT cancer.

In addition to HP1a, both dADD1a and dADD1b proteins are localized in the telomeric regions (Figure 5). In the *add1* null mutation, enrichment of HP1a at the HeT-A promoter is disrupted, while in TAS telomeric regions, there is only a half-reduction in HP1a abundance [61]. These observations suggest that a different mechanism of HP1a maintenance may be involved in different telomeric domains (HTT array and the TAS regions). Rescue experiments with overexpression of the dADD1a or dADD1b protein in *add1* null mutation background revealed that only the dADD1a protein is required to maintain correct levels of HP1a, not only at telomeres but also over chromosome arms. In contrast to HP1a, it was reported that the ADD domain may continue to bind to H3K9me3/H3K4 even if the H3Ser10 mark is present [91]. It is possible that dADD1a responds to correct levels of HP1a at telomeres where there is competition between repressive H3K9me3 and active H3K9me3/H3Ser10 histone marks, thereby maintaining the correct level of retrotransposon transcription and integration.

ATRX often colocalizes with G-quadruplexes and relaxes them [92], while stabilization of a human-specific LINE-1 element G-quadruplex structures stimulates the transposition of these elements [93,94,95]. In *Drosophila*, G-quadruplex structures have been predicted in HeT-A, TART, and TAHRE [96,97]. Thus, simultaneous inactivation of *add1* and *xnp* may result in the relaxation of such G-quadruplex structures, stimulating HeT-A retrotransposon transcription and transposition.

## 7. Evidence for the Interaction of ATRX, dXNP, and dADD1 with Insulator and Regulatory Proteins

Insulators are multifunctional elements involved in the global regulation of gene expression [98,99,100,101,102,103,104]. Two main functional features of insulators are well described, which often occur independently but cannot be completely separated in vivo. First, insulators prevent enhancer-promoter interactions if they are located between them (enhancer-blocking activity). Second, insulators located on the sides of the transgene can block the effect of surrounding repressive chromatin (barrier activity) [105,106,107,108]. The ability of insulator complexes to either directly repress or stimulate the transcription of tissue-specific genes has recently been described [109,110].

Moreover, insulators mediate intra- and inter-chromosomal interactions involved in large-scale genome organization [107,108]. They mediate contacts between regulatory elements through the formation of loops and thus participate in the organization of chromatin architecture [111,112,113,114]. Most insulator complexes are formed around one or more key DNA-binding proteins. In vertebrates, the conserved insulator protein CTCF, the MAZ factor (Myc-associated zinc-finger protein), and the BTB-containing PATZ1 protein have been described [115,116,117,118]. Cohesin and the insulator protein CTCF, responsible for folding the genome into topologically associated domains (TADs), are required to establish and maintain proper genome packaging in mammals [119]. 

It has been shown that ATRX and cohesin proteins interact with each other in the mouse neonatal brain and can bind to various imprinted loci, such as *Gtl2/Dlk1* and *H19/Igf2* [23]. At the *H19/Igf2* locus, these interactions are found in the ICR (imprinting control region) of the maternal *H19* allele, which binds CTCF insulator protein [120]. In the neonatal brain, where imprinted genes are expressed, *ATRX* inactivation caused their repression. Conversely, in the postnatal brain, in the absence of ATRX, programmed inactivation of *H19/Igf2* and other imprinted loci was blocked [121]. The absence of ATRX on the ICR of the maternal *H19* allele alters the correct positioning of nucleosomes, leading to a decrease in the level of binding of the CTCF protein and, consequently, cohesin. Distance interactions between regulatory elements at the *H19/Igf2* and *Dlk1/Gtl2* loci were also impaired [121,122]. Thus, the ATRX protein can regulate the level of binding of the insulator protein CTCF. It is possible that ATRX, by deposition of histone H3.3 in active genes and regulatory elements, helps maintain chromatin in an accessible state. However, it is not yet clear whether this effect is tissue or locus-specific.

Long noncoding RNA TERRA transcription is initiated within the subtelomeres from a promoter that contains a CTCF binding site with the peak at a position of approximately 1–1.5 kb from the TTAGGG. A strong overlap of CTCF binding sites with the cohesin subunit Rad21 has been observed [123]. ATRX peak enrichment occurred in the subtelomere at ~1.1 kb from the terminal repeats, which strongly overlaps with the CTCF binding region. Like inactivation of *ATRX*, inactivation of *CTCF* leads to a decrease in the level of RNA Pol II and cohesin recruitment and a simultaneous decrease in TERRA expression [72]. The spatial localization and overlap in the functional consequences of inactivation suggest a potential interaction between ATRX and CTCF in subtelomeric regions. However, this assumption requires experimental confirmation. In addition to TERRA transcription, CTCF may promote telomeric DNA replication by altering chromatin structure. Telomeric regions are characterized by specific histone modifications that contribute to the formation of a relatively compacted chromatin structure [124]. CTCF and cohesin binding influence histone modifications, as histone H3K4me3 levels were significantly reduced at regions adjacent to mutated CTCF-binding sites [125]. The CTCF-binding site is required for active histone H3K4me3 enrichment and may prevent the formation of heterochromatin. Spatial localization and coincidence of functional consequences of inactivation suggest a potential interplay between ATRX and CTCF at subtelomeric regions as, in many euchromatic regions, *ATRX* inactivation resulted in a dramatic reduction in histone H3.3 accumulation and the formation of less accessible chromatin [16].

It is known that CTCF exhibits a stable barrier function, separating repressive chromatin enriched with the H3K27me3 mark from open chromatin [126,127]. CTCF appears to be able to relax repressive chromatin through changes in histone molecular marks. To implement such modifications, CTCF requires additional factors such as remodeling complexes. CTCF sites involved in barrier function do not have histone repression marks but are highly enriched in the H3.3 histone variant [128,129,130]. Incorporation of histone H3.3 coincides with the binding of the insulator protein CTCF [131]. It can be hypothesized that ATRX, which (together with HIRA and DAXX) remodulates H3.3 incorporation, is involved in forming and maintaining the boundary regions established by the CTCF. This assumption is confirmed by the (direct or indirect) interaction between the insulator protein CTCF and the chaperones HIRA and Asf1 [132].

Eleven insulator proteins have been described in *D. melanogaster*, many of which (dCTCF, Su(Hw), Pita, ZIPIC, GAF, and BEAF) contain C2H2-type zinc fingers [103,133,134,135,136,137,138]. All of these are a platform for the assembly of insulator complexes containing proteins including CP190, Mod(mdg4), and HIPP1 [139,140,141,142,143,144]. Among the factors associated with telomeres in *D. melanogaster*, architectural/insulator proteins (BEAF, CP190, Pita, Z4, Chriz) and transcription factors (psq, Dip3, DREF, Adf1, etc.) have been identified [89]. Like dXNP and dADD1, many of the identified proteins (BEAF, DREF, Ken, Z4 and TRF2) act as negative regulators of TART [85,145]. Thus, the mutation in the *ken* gene is entirely reminiscent of the *add1* null mutation: it increases the expression of TART but does not change the number of its integrated copies and does not affect the activity of the HeT-A element. At the same time, mutations in *Dref*, *Trf2*, and a hypomorphic mutation in the *z4*/*putzig* gene act similarly to the *xnp*^2^/+, *add1*^2^/*add1*^2^ mutation, increasing both the expression and copy number of TART and HeT-A [61,84]. It has also been shown that a decrease in the Z4 protein in the hypomorphic allele is accompanied by a decrease in H3K9me3 and HP1a on the HeT-A promoter [84], suggesting similarities between the functions of dADD1a and Z4 in the telomere regions. Previously, dXNP was shown to repress transcription of the DREF-dependent *prn* gene through direct interaction with DREF [9]. Based on all these facts, it can be assumed that insulator proteins and dXNP/dADD1 play a tandem role in the regulation of telomere stability.

Combinations of BEAF-32, GAF, and dCTCF insulator proteins cover almost all promoters of protein-coding genes located in the pericentric chromosome regions and heterochromatic chromosome 4 in *D. melanogaster.* It was recently demonstrated that insulator proteins participate in the expression of heterochromatic genes and may facilitate their normal function. A direct impact of insulator presence on heterochromatic gene expression has been established for the GAGA factor [146]. Although the mechanism of this effect is unknown, it can be assumed that GAF-dependent insulators work in the same way in heterochromatin as in euchromatin.

Previously, H3.3 replacement peaks at the GAF-containing insulators *Mcp*, *Fab-7*, and *Fab-8 in the Bithorax* complex were observed [147]. It has been shown that GAF-containing insulators induce chromatin remodeling and direct H3.3 replacement through the interaction of GAF with the FACT (FAcilitates Chromatin Transcription) complex and the chaperone HIRA [148]. It is known that the FACT complex cannot accomplish the H3.3 replacement alone as it predominantly displaces a histone H2A-H2B pair from a nucleosome [149]. It was reported that dXNP interacts with Dre4 and SSRP1, the major components of the FACT complex [150]. Since both GAF and dXNP interact with components of the FACT complex, it is conceivable that the chromatin remodeling factor dXNP promotes the deposition of H3.3 in GAF-dependent insulators. Replacement of histone H3 with H3.3 may affect the functions of chromatin insulators. The H3.3 substitution ensures the formation of a nucleosome-free region and blocks the spread of repressive chromatin [151]. Thus, the work of insulators in the Bithorax complex and the expression of heterochromatic genes can be regulated through the joint work of insulator proteins and the dXNP protein. 

Although there is currently no evidence of direct interactions of dXNP and dADD1 with insulators, their functional interaction might be mediated by intermediary proteins. Purification of the complex containing the dADD1 protein revealed its main partners, among which were found the dSetDB1, HIPP1, Mod(mdg4), and Bonus proteins [30]. Significant co-localization was demonstrated between dSetDB1 and CP190 proteins and other insulators, BEAF and dCTCF. Such co-localization was associated with ubiquitously expressed genes [152]. Thus, it can be assumed that, in *Drosophila*, dADD1 acts with insulators to ensure the remodeling of the epigenetic status of some active heterochromatic genes, thereby making them accessible to the transcription apparatus.

The HIPP1 protein has a crotonase domain responsible for negatively regulating protein crotonylation levels [153,154]. The HIPP1 protein is localized not only in pericentric regions but also in euchromatin, where it intensively colocalized with the insulator complex proteins Su(Hw), dCTCF, CP190, and Mod(mdg4) [30]. It has been shown that in the Su(Hw)-dependent complex, HIPP1 stabilizes the binding of CP190 [139]. It is possible that HIPP1 and dADD1 cooperate at insulator sites and stabilize insulator complexes, participating in the barrier activity of insulators.

Proteins of the Mod(mdg4) family [155,156] are associated with dCTCF-, BEAF-, and Su(Hw)-dependent insulator complexes [141,157,158]. Depending on the context, these insulators can either repress or activate transcription [103,109,110,159]. It is possible that, through interaction with Mod(mdg4), the dADD1 and dXNP proteins can influence the regulatory activity of insulators.

In *Drosophila*, the Bonus protein is the only homolog of the human Trim28 family of proteins that functionally interact with ATRX [160,161,162]. Bonus protein regulates the expression of specific loci by influencing chromatin status, similar to Trim28 [163,164,165]. It would be interesting to test whether Bonus is involved in maintaining the silent state together with the dXNP and dADD1 proteins. 

## 8. Conclusions

The SWI/SNF-like chromatin remodeler ATRX is essential for maintaining genome stability and function. Mutations of the *ATRX* gene are often found in tumor development, especially in glioma, sarcoma [15], and ALT-positive tumors, although mutations in additional genes are required for ALT activation [166,167]. Increasing evidence shows that ATRX is implicated in the initiation, progression, therapy, and therapeutic resistance of cancer. Infiltrating gliomas are the most common primary malignant brain tumors, which are typically associated with a poor prognosis and low quality of life [168]. The *ATRX* is one of the twenty most frequently mutated genes in cancer and is the third most mutated gene in gliomas. In pediatric glioblastoma, somatic mutations in the H3.3-ATRX-DAXX chromatin pathway were reported in 44% of tumors, while the majority of primary malignant brain tumors in adults were associated with ATRX-dependent alternate telomere lengthening [169,170,171]. It is important to understand which mutations in ATRX are associated with different types of cancer. For example, in pediatric glioblastoma, *ATRX* mutations frequently occur near the carboxyl-terminal helicase domain, while in adult glioma mutations are evenly distributed in all genes [172,173,174]. The *ATRX* status was incorporated into the diagnostic algorithm for glioma variants combined with histology. The neuroblastoma subtype with different clinical phenotypes (resistance to traditional therapy, chronic but progressive disease course and older age at diagnosis) has been identified by *ATRX* mutations [175]. Particular DNA damage repair defects were frequently observed at ATRX deficiency in this neuroblastoma subtype [176]. The understanding of ATRX molecular functions will provide discoveries of potential cancer treatments.

**Figure 6:**
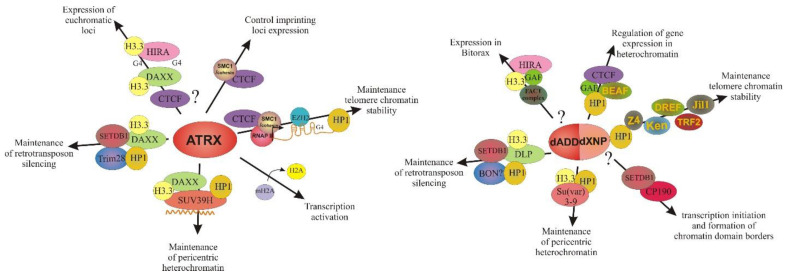
Comparison the multiple roles of ATRX and dXNP/dADD1 proteins. The scheme represents functions of ATRX on the left and dXNP/dADD1 on the right. The arrows indicate the main regulatory processes in which ATRX and dXNP/dADD1 proteins are (or may be) involved. Hypothetical participation is indicated by a question mark (“?”). In both schemes, additional proteins that participate in these pathways are indicated. Orange lines represent small ncRNA and TERRA RNA which forms G-quadruplex (G4).

The ATRX protein, through the ADD domain and the HP1a protein, binds to heterochromatic regions enriched in H3K9me3 [177]. The ATRX/DAXX complex deposits histone H3.3 in repeat regions, promoting the formation of repressive chromatin [18]. However, by acting at specific loci, ATRX can promote the formation of “open” chromatin, stimulating transcription [16]. Given the diverse roles that ATRX plays in critical cellular processes, it is clear that its contribution to genomic function is currently underestimated. To better understand how ATRX functions in various cellular processes, it is necessary to study the functioning of ATRX-associated complexes. However, in vivo, studies of native protein complexes in mammals are hampered by limitations in genetic methods and the complexity of biochemical methods. A better understanding of the mechanisms of action of ATRX may be facilitated by studying its ortholog in *Drosophila*, which is organized from two proteins, dADD1 and dXNP. Like ATRX, these proteins participate in many different and seemingly unrelated regulatory contexts. For dADD1 and dXNP, both heterochromatic and euchromatic localization are shown [28,31]. As part of various complexes, dADD1 and dXNP can regulate the transcription of both heterochromatic and euchromatic loci, maintaining the balance between active and repressive chromatin (Figure 6) [31,49,178]. 

The question arises: what allows these proteins to perform such different functions? Their diverse activity is likely explained by the presence of many protein partners that can interact directly or indirectly as part of common complexes with ATRX in humans, while their orthologs interact with dADD1 and dXNP in *Drosophila*. In *Drosophila* XNP protein is represented by two isoforms and only one of them (XNP-L) interacts with partner protein ADD1, while another one (XNP-S) seems to have independent localization. XNP-L may recruit to the chromatin by three isoforms of ADD1 protein. These isoforms have different C-terminal regions, which may be implicated in diverse regulatory processes. The ADD1a isoform represses telomeres transposon activity and maintains the HP1a level not only at telomeres but also over chromosome arms. Most likely, ADD1a does not have pronounced C-terminal domains involved in transcription repression. In contrast, little is known about ADDb and ADDc isoforms that have C-terminal regions enriched in three MADF domains. These domains may be implicated either in DNA binding or in the recruiting of partner proteins with diverse consequences for transcriptional activity. Such partners may be insulators or other regulatory proteins. It has been shown that the well-known mammalian insulator protein, CTCF, is localized with ATRX in subtelomeric regions and in regulatory regions of imprinted loci. It also affects histone modifications and chromatin structure [72,123]. Many insulator proteins have been described in *Drosophila*. Much data have been obtained on their role in regulating the expression and integration of telomeric transposons, histone H3 methylation, and histone H3.3 deposition. In the same way as dADD1 and dXNP, insulator proteins are localized on telomeres and in pericentric heterochromatin and regulate the transcription of heterochromatic genes [84,85,89,145]. In addition, many insulator proteins have zinc finger domains through which they bind DNA [133]. It can be assumed that direct or indirect interactions between insulator proteins and dADD, dXNP, or ATRX attract these remodeling factors to chromatin not marked by H3K9me3 and H3K4 and allow them to participate in the work of euchromatic complexes along with regulatory proteins. However, at present, there is only indirect evidence of interactions between insulator proteins and ATRX, dADD1/dXNP. The mechanism of such interactions requires further study.
